# On the Effects of Tincture of Colchicum in Gout

**Published:** 1815-04

**Authors:** John Lignum

**Affiliations:** Member of the Royal College of Surgeons in London. Manchester


*For the Medical and Physical Journal.*

### *On the Effects of Tincture of Colchicum in Gout;* by by Mr. J. Lignum, Jun.

IN your valuable Journal, No. 187, I observe a difference of opinion between Dr. Sutton and Mr. Want on the *modus operandi* of the Tinctura Colchici for the cure of gout. The following case strengthens Mr. Want's argument on the effects of that medicine in gout, and evidently proves it was not from the violent purgative qualities of the Tinct. Colchici that the cure was effected.

On the 14th of December last, I was requested to visit a lady 53 years of age. I found her attacked with the gout in the right foot; the ancle much swelled and inflamed, attended with considerable pain. She informed me it was the fifth attack. The paroxysms generally last from three to four weeks. I ordered her

℞. Hyd. Subm. gr. viij.

Pulv. Jalapii, gr. x.

Pulv. Scammon. gr. iv. s. q.

M. ft. Pilulæ No. iij. stat. sum.

Dec.

Dec. 15th.—9 A. M. Has had several very copious offensive stools; in other respects no alteration.

℞. Magnesiæ Vitriolat. ℥j.

Antim. Tart. gr. ¼.

Infus. Sennæ, ℥ij. M. ft. haust. stat. sum.

6 P. M. Has had several copious stools, still offensive; pain not abated.

℞. Hyd. Subm. gr. vj.
Aloes, gr. vj. M. ft. Bolus stat. sumend.

16th. Bowels well opened; stools of a more natural appearance; pain unabated.

℞. Haust. Cathartic ut antea.

17th.—Stools quite natural; pain not diminished. This day I determined to try the Tinctura Colchici. I sent her three draughts, each containing two drachms of the tincture, and desired one to be taken every six hours.

18th.—I was astonished to find my patient down stairs: she has had no stool since taking the draughts; is nearly free from pain. Rep. Haust. ut antea.

19th.—Quite free from pain; no alvine evacuation. Rep. Haustus.

20th.—Still free from pain; has had two watery stools, which she compares to coffee grounds.

21st.—Bowels regular. From this time I have given her no medicine, and she is now quite well. I attribute the cure to the colchicum, not for its purgative qualities. I shall still be an advocate for clearing the primæ viæ prior to using the colchicum, since I have read and seen its effects. I shall not implicitly trust to Epsom salts, aloetic purgatives, calomel, jalap, or gamboge; but look upon them as auxiliaries only to the grand specific. JOHN LIGNUM, Jun.

*Member of the Royal College of Surgeons in London.*

*Manchester; Feb.* 16, 1815.

